# Cross-Sectional Study on Self-Perception of Dento-Facial Asymmetry

**DOI:** 10.3390/medicina60081291

**Published:** 2024-08-10

**Authors:** Alexandra-Nina Botezatu, Eduard Radu Cernei, Georgeta Zegan

**Affiliations:** Faculty of Dental Medicine, “Grigore T. Popa” University of Medicine and Pharmacy Iasi, 16 Universitatii Str., 700115 Iasi, Romania; alexandra.botezatu@yahoo.com (A.-N.B.); georgetazegan@yahoo.com (G.Z.)

**Keywords:** facial symmetry, self-perception, dento-facial asymmetry, dental asymmetry

## Abstract

*Background and Objectives*: Facial symmetry is a key component of facial beauty and attractiveness. However, perfect symmetry is rare, and slight asymmetries, also known as natural asymmetries, are common and contribute to the uniqueness of each face. The perception of facial asymmetry varies among individuals and can be influenced by several factors. This study aimed to investigate the self-perception of dento-facial asymmetry among a sample of Romanian individuals, focusing on their awareness, the extent to which it bothers them, and their desire for correction. *Materials and Methods*: A cross-sectional analytical study was conducted with 283 participants from Romania between January and February 2024. Participants completed a questionnaire designed to assess their self-perception of facial asymmetry and socio-demographic characteristics. The questionnaire included 10 questions on self-perception of facial asymmetry and 8 questions on socio-demographic data. Statistical analysis was performed using SPSS 26.0, and the Pearson Chi-square test was used for comparative analysis. *Results*: The sample was predominantly female (75.3%) with an average age of 32.24 years. Most participants were from urban areas (80.6%) and had university degrees (58.7%). About 28.7% of participants observed facial asymmetry, with dental asymmetry being the most frequently reported, followed by asymmetries in the eyebrows and eyelids. The right side of the face was more commonly perceived as asymmetric. Although 24.4% of participants were bothered by their asymmetry, 39.2% expressed a desire to correct it. *Conclusions*: One-third of participants identified dento-facial asymmetry, with the dental level being the most reported. A significant portion of participants expressed a desire to correct their asymmetries, highlighting the importance of understanding self-perception in the context of facial aesthetics. This study underscores the subjective nature of facial asymmetry perception and the varying thresholds for what is considered bothersome or in need of correction.

## 1. Introduction

Facial symmetry represents a state of balance, a correspondence of facial features in terms of size, shape, and position relative to the mid-sagittal plane, being a key component of facial beauty and attractiveness [1]. Perfect symmetry, whether of the face or body, is extremely rare [2]. According to Srivastava et al. [3], there is no perfect bilateral symmetry of a living body. Bishara et al. [4] consider perfect body symmetry a theoretical concept. The human face is no exception, and bilateral structures exhibit a small, often imperceptible, degree of asymmetry [5]. This type of asymmetry is referred to as relative symmetry, subclinical asymmetry, slight asymmetry, or natural asymmetry [2,6]. According to Ferrario et al. [6], facial asymmetry appears to be an intrinsic feature of the human face. Thus, facial asymmetry can also be found in faces perceived as beautiful [1,7,8]. According to Bishara et al. (1994) [4], Sandor et al. (2007) [9], and Andrade et al. (2021) [10], this small and variable degree of asymmetry confers uniqueness and individuality to each human face.

In the literature, various criteria for classifying facial asymmetry can be found. These include the etiological classification of asymmetry presented by Cheong and Lo (2011) [11], the structural classification described by Bishara et al. (1994) [4], and the architectural classification of asymmetry described by Pirttiniemi (1999) [12].

The reported prevalence of facial asymmetry varies significantly in the specialized literature, ranging from 12% [13] to 34% [14] in the United States, around 23% in Europe [15], 21% in Asia [16], and 32% in South America [17]. In Romania, the prevalence of dento-facial asymmetry is 4.7% [18].

Within dento-facial asymmetries, those present in the lower third of the face account for 74%, followed by asymmetries in the middle third at 36%, and those in the upper third of the face represent only 5% of the total dento-facial asymmetries [14]. Chin deviation appears to be the most common manifestation of dento-facial asymmetry [19,20]. This distribution of asymmetry within the facial thirds could be due to the maxilla’s rigid interconnection with adjacent cranial structures considered stable, while the mandible is practically a mobile system with a longer growth period [2,21]. The appearance of asymmetry at the mandibular level may be due to the adaptation of the anatomical structure to deviations that occur during functional activities, causing modifications and remodeling of the condyle, glenoid fossa, and mandibular body [22].

Since the degree of asymmetry of a face can range from very small, almost imperceptible, to very large, attempts have been made to find or define a threshold or a limit between normal and abnormal [4]. Specialists and patients may have different views on this limit, often being subjective. A trained eye of a specialist can detect a small difference between the two hemifaces, while the patient either does not detect the asymmetry, detects it but does not consider it bothersome, considers a very small degree of asymmetry unacceptable, or is guided by how others perceive them [23].

The objective of our research was to study the perception of the participants on their own face and to determine if they consider themselves to have a dento-facial asymmetry. Also, we wanted to see if the asymmetry was considered to be unacceptable by the participants and if the participants wanted to seek treatment for it.

## 2. Materials and Methods

### 2.1. Study Design

A cross-sectional analytical study was conducted on a sample of 283 participants from Romania. The study was carried out from January to February 2024. 

### 2.2. Study Phases

This study focused on the following phases: (1) description of the study sample characteristics and evaluation of self-perception of dento-facial asymmetry, and (2) comparative study of self-perception of dento-facial asymmetry based on the socio-demographic characteristics of the respondents.

### 2.3. Study Instrument

To achieve this study objectives, we used a questionnaire consisting of two sections. The questionnaire was designed before the actual study began due to the absence of an adequate questionnaire in the specialized literature for our research purpose.

The first section included 10 questions about self-perception of facial asymmetry and the anatomical level at which it can be found. Additionally, study participants were asked if they were bothered by facial asymmetry and if they wished to correct it. Participants were asked to look in the mirror and self-identify, through visual evaluation, the dento-facial asymmetries they believed they had. To evaluate the attitudes or perceptions of study participants to the questionnaire questions, a 5-point Likert scale was used, with responses ranging from strong agreement to strong disagreement, with intermediate positions being agreement, neutral, and disagreement.

The second section included 8 questions about general socio-demographic data. The questionnaire was created using the Google Forms web application and was disseminated online via email and messaging applications such as WhatsApp and Messenger by sending a link that the participants accessed.

### 2.4. Statistical Analysis

Participants’ responses were stored in a database using Microsoft Excel. Statistical analysis was performed using SPSS 26.0 (IBM, Armonk, NY, USA) for Windows. The following statistical parameters were calculated: arithmetic mean, standard deviation, standard error of the mean, and median. The Pearson Chi-square test was used for comparative analysis of results based on demographic variables. A *p*-value below 0.05 was considered statistically significant. For the study of correlations between physical activity level, incorrect posture, presence of dento-facial asymmetry, and existence of body asymmetry, Spearman’s rho correlation coefficients were calculated, considering the qualitative–ordinal nature of the analyzed variables.

### 2.5. Ethical Considerations

Prior to the study, ethical approval was obtained from the Research Ethics Committee of “Grigore T. Popa” University of Medicine and Pharmacy in Iasi, nr.149/3.02.2022. All study participants signed an informed consent form approved by the Research Ethics Committee of “Grigore T. Popa” University of Medicine and Pharmacy in Iasi.

## 3. Results

### 3.1. Characteristics of the Sample

The characteristics of the sample are presented in Table 1. The respondents to the questionnaire were predominantly female (75.3%) and relatively young; thus, the average age of the patients in the sample was 32.24 ± 10.192 years. Women were slightly older than men, with an average age of 33.26 ± 10.468 years compared to 29.16 ± 8.662 years. Most patients came from urban areas (80.6%); additionally, more than half of them (58.7%) had university degrees, half were single (51.2%), and almost half were married (43.1%). Furthermore, 63.9% of patients reported having average or above-average income. Regarding professions, most patients were employed, with 18.7% working in the medical field; also, a significant proportion of patients were students, accounting for 29.0%, with nearly half of them being general medicine students (13.8%). Just over 60% of patients (61.1%) lived on the 1st–2nd or 3rd–4th floors.

### 3.2. Evaluation of Self-Perception of Dento-Facial Asymmetry

First, patients were asked if they generally observed any dento-facial asymmetry in their face. Only a quarter of them agreed with this phenomenon (28.7%), with 17.3% localizing the asymmetry on the right side and 14.8% on the left side (Table 2).

Self-perceived asymmetries are presented in the order of frequency reported by patients: dental asymmetry, eyebrow asymmetry, eyelid asymmetry, nasal asymmetry, mouth asymmetry, cheek asymmetry, and chin asymmetry, with chin asymmetry being perceived by the fewest patients (Table 3).

Only 24.4% of them considered that these asymmetries bother them, with the majority being neutral (41.0%) or disagreeing with this statement (34.7%). However, a higher percentage of patients, almost double, declared that they would like to correct their observed dento-facial asymmetry (39.2%); a third were neutral (34.3%) and just over a quarter partially or totally disagreed (26.5%) (Table 4).

### 3.3. Comparative Study of Self-Perception of Dento-Facial Asymmetry Based on Socio-Demographic Characteristics of Respondents

Significant statistical differences were also observed between genders regarding the presence of observed dento-facial asymmetries; thus, a higher proportion of women noticed such asymmetries (32.9%) compared to only 15.8% of men. Similarly, nearly double the proportion of women than men (47.4% vs. 25.8%) reported such asymmetries in their eyebrows. Other significant statistical differences were observed in dental asymmetries—although relatively similar percentages of women and men expressed full or partial agreement about the presence of these asymmetries (46.9% vs. 48.6%), the proportion of women who strongly agreed was almost four times higher than the similar proportion of men (16.9% vs. only 4.3%). No other significant statistical differences were observed between genders, although it is worth noting that 32.9% of women considered their eyelids to be asymmetric compared to only 25.7% of men, and 24.4% of women considered their mouth corners to be asymmetric compared to only 20.0% of men. In total, 27.7% of women were bothered by the dento-facial asymmetries they had observed, compared to only 14.3% of men, but this time the difference between the genders was not statistically significant, and the percentages of patients who wanted to correct these asymmetries were similar for both women and men, around 40% (Table 5).

Overall, no significant statistical differences were observed between the two age groups regarding the percentages of patients who observed dento-facial asymmetries. Thus, 28.4% of patients under 30 noticed such asymmetries, similar to 28.9% of those over 30. However, a significantly higher percentage of younger patients observed eyebrow asymmetries compared to patients over 30 (48.0% vs. 35.6%). No other significant statistical differences were observed, although it is worth noting that younger patients reported slightly higher percentages of asymmetries in the eyelids (33.1%), cheeks (23.0%), and chin (12.9%), while patients over 30 reported higher percentages of dental asymmetry (50.3%) compared to younger patients (44.6%). Regardless of age group, about a quarter of patients were bothered by the dento-facial asymmetries they observed, and the percentage of those who wanted to correct these asymmetries was higher in younger patients (41.2%) compared to patients over 30 (37.0%) (Table 6).

In total, 40.0% of rural patients believed they had dento-facial asymmetries, compared to only 25.9% of urban patients, a significant and statistically important difference. Slightly higher percentages of rural patients localized these asymmetries in the eyelids (34.5%), mouth corners (30.9%), and teeth (52.8%), but these differences were not statistically significant compared to similar percentages observed in urban areas. It is also noted that the proportion of urban patients who were bothered by their dento-facial asymmetries was slightly higher than the similar proportion of rural patients (25.5% vs. 20.0%), but the percentages of patients who would like to correct these asymmetries were very close in both environments (40.0% in rural areas and 39.0% in urban areas) (Table 7).

No statistically significant differences were observed among the three categories of patients identified by their education level regarding the observation of dento-facial asymmetries. However, a significant statistical difference was noted in the self-assessments related to the corners of the mouth, with this facial asymmetry being reported by 22.9% of patients with university education and 17.6% of those with postgraduate education (*p* = 0.044).

The same observation applied to the self-perception of dento-facial asymmetry based on the marital status of the respondents, with discrepancies between the four categories of respondents that did not reach statistical significance.

The most respondents who reported observing dento-facial asymmetries belonged to the category with average income (33.7%); this percentage was significantly higher than the similar percentages observed in the other categories of respondents: only 26.5% of those without income, 23.5% of those with minimum income, and 25.7% of those with above-average income (*p* = 0.002).

Another significant statistical difference in the sample related to dental asymmetry, with 45.6% of respondents without income, 44.1% of those with minimum income, and 43.2% of those with above-average income observing that they have asymmetric teeth (*p* = 0.028).

The only significant statistical difference in the sample at the level of dento-facial asymmetries was observed in the teeth; thus, 66.7% of self-employed or unemployed respondents considered their teeth to be asymmetric, compared to only 40.4% of employees, 42.7% of students, and 55.9% of patients with other professions (*p* = 0.023).

However, significant statistical differences were observed between the four categories of respondents identified by their housing type regarding the presence of dento-facial asymmetries. Thus, a third of patients living on the ground floor (33.8%) believed they have such asymmetries, compared to 24.1% of those living on the 1st–2nd floors, 30.4% of those living on the 3rd–4th floors, and 28.9% of those living on upper floors (*p* = 0.027). Similar percentages of patients living on intermediate floors ranged between 30 and 35%, with the recorded difference being statistically significant (*p* = 0.013).

## 4. Discussion

Peck et al. (1991), following a study on postero-anterior cephalograms, stated that the stability of the facial complex is higher in areas closer to the skull, presenting small degrees of asymmetry. They reported the greatest deviations at the mandibular level, moderate deviations at the zygomatic level, and minor deviations in the orbital area [24]. According to Haraguchi et al. (2008), dento-facial asymmetry is equally distributed among skeletal classes I, II, and III [21]. However, other studies have shown a greater association of dento-facial asymmetry with skeletal class III [22] or a lower association with skeletal class II [14]. Regarding the distribution of asymmetry at the frontal level, studies support the predominant involvement of the left hemiface [2,6,21,25]. This can be attributed to greater growth of the right hemiface, consistent with the larger dimensions of the skull and brain on the right side [2,6]. Rohrich et al. (2016), in a study conducted on photographs, showed that the chin and nose are deviated towards the smaller hemiface [26]. As for the distribution of facial asymmetry by gender, it appears to be equal between women and men according to studies conducted so far [2,5,22,27]. However, these results sometimes differ, and depending on the characteristics of the studied group and the method used for evaluating facial asymmetry, there are also studies that have found higher values for asymmetries in the upper face [25] and the middle face [7,26,28].

Chatrath et al. (2007), in a study conducted on a group of patients seeking rhinoplasty, found a very high percentage, 90%, of significant dento-facial asymmetries [29]. MacDonald et al. (2012), in a study conducted on a group of patients seeking blepharoplasty, reported a 75% percentage of patients with asymmetry in the eyebrows and/or eyelids greater than 2 mm [30].

Identifying a perception threshold for dento-facial asymmetry is a complex process influenced by numerous factors, including the observer’s level of training regarding dento-facial asymmetry, gender, the importance the observer places on dento-facial asymmetry, the part of the face where the asymmetry manifests, and the facial element affected by asymmetry [22]. Naini et al. (2012), in a study conducted to determine how the severity of facial asymmetry influences a person’s attractiveness, showed that for deviations of the chin less than 5 mm, facial asymmetry is negligible for patients and they do not wish to correct it. However, for deviations greater than 5 mm, patients’ desire to seek surgical treatment to correct the asymmetry increases. Additionally, the same study identified the main factors influencing the desire of patients with dento-facial asymmetry to seek surgical treatment: age, gender, and ethnicity [31]. Thus, as patients age, their desire for surgical treatment of dento-facial asymmetry decreases, men are less likely than women to seek surgical methods, and Caucasians are more likely to seek surgical treatment [31].

According to Wang et al. (2017), knowing the limit at which acceptable asymmetry becomes unacceptable for the patient is of utmost importance for pre-surgical evaluation, as it can improve the proposed treatment plan and, consequently, patient satisfaction by establishing realistic expectations from the treatment [23].

In the context of a stimulus that draws a person’s attention to themselves (looking at their image in the mirror), that person becomes more self-aware and more attentive to the differences between their ideal self and the real self (seen in the mirror) [32]. This way, the person can notice their own dento-facial asymmetry. On the other hand, Lu and Bartlett (2014) believe that a person looking at themselves in the mirror and seeing their inverted image may not be able to accurately assess their expressiveness and attractiveness, with the perception of their own facial asymmetry being affected by the inverted image [33].

Another limitation of this study is represented by the sample used, which is not representative for any population.

In the conducted study, only a quarter of the patients reported having dento-facial asymmetry; however, it is interesting to note that once they became aware of the asymmetry, patients were able to locate it correctly. Approximately half localized it on the right side and about half on the left side—the two percentages combined being almost equal to the percentage of patients who generally noticed asymmetries and practically had no doubts in specifying it correctly. These results confirm the data provided by Chatrath et al. (2007), who reported that about half of the patients seem to be aware of the presence of dento-facial asymmetry [29]. On the other hand, MacDonald et al. (2012), in a study conducted on a group of patients where approximately three-quarters of them had facial asymmetries, emphasized that no patient brought up asymmetry as a reason for seeking medical attention, suggesting that they were not aware of its presence [30].

The most frequently reported asymmetry by patients is at the dental level. Thus, almost half of them believe they have asymmetric teeth, predominantly on the right side, while less than a quarter of patients locate the same asymmetry on the left side. This is interesting because, according to studies conducted so far, deviations of the maxillary midline up to 4 mm or sometimes even larger seem to go unnoticed by non-specialists [34]. On the other hand, asymmetry due to different shapes of the central maxillary incisors is noticed by non-specialists even with a 0.5 mm difference between the lengths of the vestibular faces of the two central maxillary incisors, and gingival contour asymmetry at the maxillary frontal area is noticed at differences of 1.5 mm [35]. These findings suggest that when patients self-evaluated dental asymmetry, they considered not only the midline deviation but also other determining factors of smile aesthetics.

Next in the order of reported asymmetries are the eyebrows, with less than half of the patients considering their eyebrows asymmetric, and less than a quarter thinking that the right eyebrow is lower. These results are consistent with those reported by Perumal (2018), who found a significant percentage of patients with eyebrow asymmetry, with most presenting a lower right eyebrow in the studied group [36]. 

Following in the order of frequency of asymmetries are the eyelids, with more than a quarter of patients considering them asymmetric, most believing the right eyelid is lower. This result is somewhat surprising, as the literature highlights that patients are often unaware of asymmetry in the periocular area [30,37]. 

A quarter of respondents consider their nose to be deviated to one side, with most reporting a deviation to the right side. This result is also interesting. In specialized studies, the nose, being centrally located on the face, is immediately noticeable, and Rohrich et al. (2017) reported a high percentage of patients who consider their nose to be deviated [26]. 

Less than a quarter of patients believe their mouth corners are asymmetric, with most observing the asymmetry on the right side. Yamamoto et al. (2015) also reported that lip asymmetries are less common in the population [38]. 

Less than a fifth of respondents believe they have a flatter cheek, with the majority indicating the right cheek. According to Zhang et al. (2023), non-specialists tend to confuse cheek asymmetry with mandibular asymmetry [39]. 

Patients most rarely observed asymmetries at the chin level. This aspect contradicts findings in the specialized literature. Most studies show that the highest degree of facial asymmetry is found in the lower third of the face, especially at the chin level, with this type of asymmetry being the easiest to detect by non-specialists [39].

This is the hierarchy of facial regions considered asymmetric from the patients’ perspective. It is also notable that in all cases, patients predominantly notice asymmetry on the right side of the face. In the specialized literature, on the contrary, left hemiface asymmetry is more common [6]. Since this study is one of patient self-evaluation, it is possible that participants did not accurately evaluate their face due to the inverted mirror image.

On the other hand, patients are not very bothered by the dento-facial asymmetries they have observed. Thus, only a quarter of them believe these asymmetries bother them, with the majority of patients being neutral or disagreeing with this statement. Conversely, a higher percentage of patients, nearly double, declare that they would like to correct the observed dento-facial asymmetry; a third are neutral, and just over a quarter partially or totally disagree.

## 5. Conclusions

Based on this study, we conclude the following:

One-third of the participants identified the presence of a dento-facial asymmetry;

The most frequently reported dento-facial asymmetry by participants is at the dental level, followed by asymmetry in the eyebrows and eyelids;

According to the observations of study participants, the right side is more affected by facial asymmetry;

A small portion of study participants are bothered by the presence of dento-facial asymmetry, but twice as many patients declare a desire to correct it.

## Figures and Tables

**Table 1:** Demographic characteristics of the investigated sample.

		n	%
Gender	Female	213	75.3
	Male	70	24.7
Age group	18–30 years	148	52.3
	Over 30 years	135	47.7
Environment	Rural	55	19.4
	Urban	228	80.6
Education	High School	43	15.2
	University	166	58.7
	Postgraduate	74	26.1
Marital status	Married	122	43.1
	Divorced	13	4.6
	Single	145	51.2
	Widowed	3	1.1
Economic status	No income	68	24.0
	Minimum income	34	12.0
	Average income	107	37.8
	Above average income	74	26.1
Profession	Medical employee	53	18.7
	Military/Police	3	1.1
	Art and culture	4	1.4
	IT	25	8.8
	Office worker	14	4.9
	Freelancer	15	5.3
	Unemployed	3	1.1
	Student	30	10.6
	Dentistry student	13	4.6
	General medicine student	39	13.8
	Other	84	29.7
Residence	Ground floor	65	23.0
	1st–2nd floor	104	36.7
	3rd–4th floor	69	24.4
	Upper floor	45	15.9
Total		283	100.0

**Table 2:** Evaluation of self-perception of dento-facial asymmetry.

	Strongly Agree		Agree		Neutral		Disagree		Strongly Disagree		Total	
	n	%	n	%	n	%	n	%	n	%	n	%
1. Do you observe dento-facial asymmetry?												
A. Yes	18	6.4	63	22.3	97	34.3	67	23.7	38	13.4	283	100.0
B. The right side is smaller	9	3.2	40	14.1	96	33.9	95	33.6	43	15.2	283	100.0
C. The left side is smaller	4	1.4	38	13.4	100	35.3	96	33.9	45	15.9	283	100.0

**Table 3:** Self-perceived asymmetries reported by patients.

	Strongly Agree		Agree		Neutral		Disagree		Strongly Disagree		Total	
	n	%	n	%	n	%	n	%	n	%	n	%
2. Do you have asymmetric eyebrows?												
A. Yes	40	14.1	79	27.9	57	20.2	72	25.4	35	12.4	283	100.0
B. The right eyebrow is lower	17	6.0	49	17.3	76	26.9	99	35.0	42	14.8	283	100.0
C. The left eyebrow is lower	21	7.4	34	12.0	77	27.2	104	36.7	47	16.6	283	100.0
3. Do you have asymmetric eyelids?												
A. Yes	27	9.5	61	21.6	70	24.8	84	29.7	41	14.5	283	100.0
B. The right eyelid is lower	16	5.7	39	13.8	79	27.9	105	37.1	44	15.5	283	100.0
C. The left eyelid is lower	11	3.9	18	6.4	85	30.0	118	41.7	51	18.0	283	100.0
4. Is your nose deviated to one side?												
A. Yes	22	7.8	50	17.7	67	23.7	96	33.9	48	17.0	283	100.0
B. Deviated to the right	9	3.2	36	12.7	72	25.5	110	38.9	56	19.8	283	100.0
C. Deviated to the left	7	2.5	27	9.5	78	27.6	114	40.3	57	20.1	283	100.0
5. Is your chin deviated to one side?												
A. Yes	3	1.1	26	9.2	68	24.0	118	41.7	68	24.0	283	100.0
B. Deviated to the right	2	0.7	19	6.7	72	25.4	122	43.1	68	24.0	283	100.0
C. Deviated to the left	2	0.7	14	4.9	72	25.4	127	44.9	68	24.0	283	100.0
6. Is one cheek flatter?												
A. Yes	9	3.2	47	16.6	72	25.4	106	37.5	49	17.3	283	100.0
B. On the right side	7	2.5	29	10.2	80	28.3	116	41.0	51	18.0	283	100.0
C. On the left side	4	1.4	18	6.4	81	28.6	125	44.2	55	19.4	283	100.0
7. Are the corners of your mouth asymmetric?												
A. Yes	17	6.0	49	17.3	78	27.6	93	32.9	46	16.3	283	100.0
B. The right corner is lower	4	1.4	28	9.9	87	30.7	112	39.6	52	18.4	283	100.0
C. The left corner is lower	6	2.1	21	7.4	89	31.4	112	39.6	55	19.4	283	100.0
8. Do you have asymmetric teeth?												
A. Yes	39	13.8	95	33.6	64	22.6	55	19.4	30	10.6	283	100.0
B. On the right side	20	7.1	69	24.4	92	32.5	72	25.4	30	10.6	283	100.0
C. On the left side	19	6.7	47	16.6	102	36.0	83	29.3	32	11.3	283	100.0

**Table 4:** Attitude towards asymmetry.

	Strongly Agree		Agree		Neutral		Disagree		Strongly Disagree		Total	
	n	%	n	%	n	%	n	%	n	%	n	%
9. Does your dento-facial asymmetry bother you?	17	6.0	52	18.4	116	41.0	61	21.6	37	13.1	283	100.0
10. Do you want to correct your dento-facial asymmetry?	29	10.2	82	29.0	97	34.3	43	15.2	32	11.3	283	100.0

**Table 5:** Comparative evaluation of dento-facial asymmetries by gender.

		Gender				Pearson Chi-Squared Test
		Female		Male		
		n	%	n	%	
Dento-Facial Asymmetries:						
1. Do you observe dento-facial asymmetry?	Strongly Agree	16	7.5%	2	2.9%	Chi^2^ = 16.988
	Agree	54	25.4%	9	12.9%	*p* = 0.002 *
	Neutral	78	36.6%	19	27.1%	
	Disagree	41	19.2%	26	37.1%	
	Strongly Disagree	24	11.3%	14	20.0%	
2. Do you have asymmetric eyebrows?	Strongly Agree	38	17.8%	2	2.9%	Chi^2^ = 14.052
	Agree	63	29.6%	16	22.9%	*p* = 0.015 *
	Neutral	39	18.3%	18	25.7%	
	Disagree	49	23.0%	23	32.9%	
	Strongly Disagree	24	11.3%	11	15.7%	
3. Do you have asymmetric eyelids?	Strongly Agree	23	10.8%	4	5.7%	Chi^2^ = 2.476
	Agree	47	22.1%	14	20.0%	*p* = 0.780
	Neutral	52	24.4%	18	25.7%	
	Disagree	62	29.1%	22	31.4%	
	Strongly Disagree	29	13.6%	12	17.1%	
4. Do you have a deviated nose?	Strongly Agree	19	8.9%	3	4.3%	Chi^2^ = 2.753
	Agree	36	16.9%	14	20.0%	*p* = 0.600
	Neutral	51	23.9%	16	22.9%	
	Disagree	69	32.4%	27	38.6%	
	Strongly Disagree	38	17.8%	10	14.3%	
5. Do you have a deviated chin?	Strongly Agree	2	0.9%	1	1.4%	Chi^2^ = 0.559
	Agree	19	8.9%	7	10.0%	*p* = 0.967
	Neutral	53	24.9%	15	21.4%	
	Disagree	89	41.8%	29	41.4%	
	Strongly Disagree	50	23.5%	18	25.7%	
6. Do you have a flatter cheek?	Strongly Agree	7	3.3%	2	2.9%	Chi^2^ = 0.578
	Agree	34	16.0%	13	18.6%	*p* = 0.965
	Neutral	56	26.3%	16	22.9%	
	Disagree	80	37.6%	26	37.1%	
	Strongly Disagree	36	16.9%	13	18.6%	
7. Are the corners of your mouth asymmetric?	Strongly Agree	14	6.6%	3	4.3%	Chi^2^ = 3.591
	Agree	38	17.8%	11	15.7%	*p* = 0.464
	Neutral	63	29.6%	15	21.4%	
	Disagree	66	31.0%	27	38.6%	
	Strongly Disagree	32	15.0%	14	20.0%	
8. Do you have asymmetric teeth?	Strongly Agree	36	16.9%	3	4.3%	Chi^2^ = 11.705
	Agree	64	30.0%	31	44.3%	*p* = 0.020 *
	Neutral	52	24.4%	12	17.1%	
	Disagree	41	19.2%	14	20.0%	
	Strongly Disagree	20	9.4%	10	14.3%	
9. Does your dento-facial asymmetry bother you?	Strongly Agree	16	7.5%	1	1.4%	Chi^2^ = 8.351
	Agree	43	20.2%	9	12.9%	*p* = 0.080
	Neutral	86	40.4%	30	42.9%	
	Disagree	45	21.1%	16	22.9%	
	Strongly Disagree	23	10.8%	14	20.0%	
10. Do you want to correct your dento-facial asymmetry?	Strongly Agree	21	9.9%	8	11.4%	Chi^2^ = 1.069
	Agree	63	29.6%	19	27.1%	*p* = 0.899
	Neutral	74	34.7%	23	32.9%	
	Disagree	33	15.5%	10	14.3%	
	Strongly Disagree	22	10.3%	10	14.3%	
Total		213	100.0%	70	100.0%	

* Significant statistical difference (*p* value < 0.05)

**Table 6:** Comparative evaluation of dento-facial asymmetries by age group.

		Age Group				Pearson Chi-Squared Test
		18–30 Years		Over 30 Years		
		n	%	n	%	
Dento-Facial Asymmetries:						
1. Do you observe dento-facial asymmetry?	Strongly Agree	9	6.1%	9	6.7%	Chi^2^ = 0.488
	Agree	33	22.3%	30	22.2%	*p* = 0.975
	Neutral	52	35.1%	45	33.3%	
	Disagree	33	22.3%	34	25.2%	
	Strongly Disagree	21	14.2%	17	12.6%	
2. Do you have asymmetric eyebrows?	Strongly Agree	26	17.6%	14	10.4%	Chi^2^ = 9.958
	Agree	45	30.4%	34	25.2%	*p* = 0.041 *
	Neutral	21	14.2%	36	26.7%	
	Disagree	35	23.6%	37	27.4%	
	Strongly Disagree	21	14.2%	14	10.4%	
3. Do you have asymmetric eyelids?	Strongly Agree	17	11.5%	10	7.4%	Chi^2^ = 3.673
	Agree	32	21.6%	29	21.5%	*p* = 0.452
	Neutral	31	20.9%	39	28.8%	
	Disagree	44	29.7%	40	29.6%	
	Strongly Disagree	24	16.2%	17	12.6%	
4. Do you have a deviated nose?	Strongly Agree	8	5.4%	14	10.4%	Chi^2^ = 7.708
	Agree	31	20.9%	19	14.1%	*p* = 0.103
	Neutral	30	20.3%	37	27.4%	
	Disagree	49	33.1%	47	34.8%	
	Strongly Disagree	30	20.3%	18	13.3%	
5. Do you have a deviated chin?	Strongly Agree	2	1.4%	1	0.7%	Chi^2^ = 3.238
	Agree	17	11.5%	9	6.7%	*p* = 0.519
	Neutral	33	22.3%	35	25.9%	
	Disagree	58	39.2%	60	44.4%	
	Strongly Disagree	38	25.7%	30	22.2%	
6. Do you have a flatter cheek?	Strongly Agree	6	4.1%	3	2.2%	Chi^2^ = 3.634
	Agree	28	18.9%	19	14.1%	*p* = 0.458
	Neutral	33	22.3%	39	28.9%	
	Disagree	53	35.8%	53	39.3%	
	Strongly Disagree	28	18.9%	21	15.6%	
7. Are the corners of your mouth asymmetric?	Strongly Agree	13	8.8%	4	3.0%	Chi^2^ = 6.135
	Agree	23	15.5%	26	19.3%	*p* = 0.189
	Neutral	36	24.3%	42	31.1%	
	Disagree	50	33.8%	43	31.9%	
	Strongly Disagree	26	17.6%	20	14.8%	
8. Do you have asymmetric teeth?	Strongly Agree	16	10.8%	23	17.0%	Chi^2^ = 2.833
	Agree	50	33.8%	45	33.3%	*p* = 0.586
	Neutral	34	23.0%	30	22.2%	
	Disagree	30	20.3%	25	18.5%	
	Strongly Disagree	18	12.2%	12	8.9%	
9. Does your dento-facial asymmetry bother you?	Strongly Agree	6	4.1%	11	8.1%	Chi^2^ = 3.224
	Agree	29	19.6%	23	17.0%	*p* = 0.521
	Neutral	61	41.2%	55	40.7%	
	Disagree	30	20.3%	31	23.0%	
	Strongly Disagree	22	14.9%	15	11.1%	
10. Do you want to correct your dento-facial asymmetry?	Strongly Agree	12	8.1%	17	12.6%	Chi^2^ = 4.360
	Agree	49	33.1%	33	24.4%	*p* = 0.359
	Neutral	51	34.5%	46	34.1%	
	Disagree	19	12.8%	24	17.8%	
	Strongly Disagree	17	11.5%	15	11.1%	
Total		148	100.0%	135	100.0%	

* Significant statistical difference (*p* value < 0.05)

**Table 7:** Comparative evaluation of dento-facial asymmetries by environment.

		Environment				Pearson Chi-Squared Test
		Rural		Urban		
		n	%	n	%	
Dento-Facial Asymmetries:						
1. Do you observe dento-facial asymmetry?	Strongly Agree	5	9.1%	13	5.7%	Chi^2^ = 15.156
	Agree	17	30.9%	46	20.2%	*p* = 0.004 *
	Neutral	25	45.5%	72	31.6%	
	Disagree	6	10.9%	61	26.8%	
	Strongly Disagree	2	3.6%	36	15.8%	
2. Do you have asymmetric eyebrows?	Strongly Agree	8	14.5%	32	14.0%	Chi^2^ = 2.254
	Agree	15	27.3%	64	28.1%	*p* = 0.689
	Neutral	11	20.0%	46	20.2%	
	Disagree	17	30.9%	55	24.1%	
	Strongly Disagree	4	7.3%	31	13.6%	
3. Do you have asymmetric eyelids?	Strongly Agree	8	14.5%	19	8.3%	Chi^2^ = 4.549
	Agree	11	20.0%	50	21.9%	*p* = 0.337
	Neutral	14	25.5%	56	24.6%	
	Disagree	18	32.7%	66	28.9%	
	Strongly Disagree	4	7.3%	37	16.2%	
4. Do you have a deviated nose?	Strongly Agree	7	12.7%	15	6.6%	Chi^2^ = 3.432
	Agree	7	12.7%	43	18.9%	*p* = 0.488
	Neutral	14	25.5%	53	23.2%	
	Disagree	19	34.5%	77	33.8%	
	Strongly Disagree	8	14.5%	40	17.5%	
5. Do you have a deviated chin?	Strongly Agree	0	0.0%	3	1.3%	Chi^2^ = 5.601
	Agree	4	7.3%	22	9.6%	*p* = 0.231
	Neutral	14	25.5%	54	23.7%	
	Disagree	29	52.7%	89	39.0%	
	Strongly Disagree	8	14.5%	60	26.3%	
6. Do you have a flatter cheek?	Strongly Agree	1	1.8%	8	3.5%	Chi^2^ = 1.000
	Agree	10	18.2%	37	16.2%	*p* = 0.910
	Neutral	12	21.8%	60	26.3%	
	Disagree	22	40.0%	84	36.8%	
	Strongly Disagree	10	18.2%	39	17.1%	
7. Are the corners of your mouth asymmetric?	Strongly Agree	5	9.1%	12	5.3%	Chi^2^ = 3.521
	Agree	12	21.8%	37	16.2%	*p* = 0.475
	Neutral	13	23.6%	65	28.5%	
	Disagree	19	34.5%	74	32.5%	
	Strongly Disagree	6	10.9%	40	17.5%	
8. Do you have asymmetric teeth?	Strongly Agree	4	7.3%	35	15.4%	Chi^2^ = 6.308
	Agree	25	45.5%	70	30.7%	*p* = 0.177
	Neutral	11	20.0%	53	23.2%	
	Disagree	8	14.5%	47	20.6%	
	Strongly Disagree	7	12.7%	23	10.1%	
9. Does your dento-facial asymmetry bother you?	Strongly Agree	0	0.0%	17	7.5%	Chi^2^ = 5.822
	Agree	11	20.0%	41	18.0%	*p* = 0.213
	Neutral	26	47.3%	90	39.5%	
	Disagree	13	23.6%	48	21.1%	
	Strongly Disagree	5	9.1%	32	14.0%	
10. Do you want to correct your dento-facial asymmetry?	Strongly Agree	3	5.5%	26	11.4%	Chi^2^ = 6.349
	Agree	19	34.5%	63	27.6%	*p* = 0.175
	Neutral	18	32.7%	79	34.6%	
	Disagree	12	21.8%	31	13.6%	
	Strongly Disagree	3	5.5%	29	12.7%	
Total		55	100.0%	228	100.0%	

* Significant statistical difference (*p* value < 0.05)

## Data Availability

All the data used in this study are available on request from the corresponding author.
